# Post direct acting anti-viral agents associated primary hepatic Castleman's disease: A case report

**DOI:** 10.1016/j.amsu.2020.08.019

**Published:** 2020-08-23

**Authors:** Dina Sweed, Heba Abdelhalim, Yahya Fayed, Asmaa Mosbeh, Shimaa Kilany, Nermine Ehsan, Dina El-Azab, Mai Shalan, Thanaa Helal

**Affiliations:** a Pathology, National Liver Institute, Menoufia University, Egypt; b Radiology, National Liver Institute, Menoufia University, Egypt; c Hepatopanceatobiliary Surgery, National Liver Institute, Menoufia University, Egypt; d Hepatology, National Liver Institute, Menoufia University, Egypt; e Pathology, Ain Shams University, Egypt

**Keywords:** Case report, Castleman's disease, DAAs, Imaging, Pathology

## Abstract

Castleman's disease (CD) is a primary lymphoproliferative disorder of the lymph nodes with rare extra-nodal primary affection. Presentation of case: Here we present a case of primary hepatic CD associated with hepatocellular carcinoma (HCC). Discussion: Sixty-two years old, male received direct-acting antiviral agents (DAAs) for HCV infection. Follow up revealed sustained virologic response; however, three hepatic focal lesions were accidently discovered. Triphasic CT confirmed the HCC nature of two masses while the other mass remained undiagnosed. Surgical intervention was the treatment of choice, and pathological examination showed a fairly circumscribed mass formed of angiolymphoid hyperplasia displayed atrophic germinal center, expanded mantle zone, and variable hyalinization. The radiological evaluation of lymph nodes was unremarkable. The patient is 40 months alive after resection, with no further management advised. Conclusion: The immune-modulatory effect of DAAs may induce hepatic CD development in a cirrhotic patient, necessitating further studies. A new radiologic finding was observed in the present case in the form of vessels traversing through the lesion with no attenuation or occlusion. Pathology remains the gold standard in the diagnosis of CD.

## Highlights

• Eighteen cases of primary hepatic Castleman's disease (CD) has been reported.
• The association of post direct-acting anti-viral agents with the development of both hepatocellular carcinoma (HCC) and CD may have immune base.
• A new radiologic finding of hepatic CD in the form of vessels traversing through the lesion with no attenuation or occlusion has been encountered in this case.
• Pathology remains the gold standard in the diagnosis of CD.

## List of abbreviations

CD: Castleman disease
UCD: Unicenteric CD
MCD: Multicentric
HV: Hyaline vascular
PC: Plasma cell
HCC: Hepatocellular carcinoma
DAAs: Direct-acting antiviral agents
HCV: Hepatitis C virus
SVR: Sustained virologic response
CT: Computed tomography
GC: Germinal center
FDCs: Follicular dendritic cells
EGFR: Epidermal growth factor receptor
RS: Reed Sternberg

## 1 Introduction

Castleman disease (CD) is a lymphoproliferative disease reported first in 1954 as a localized lymph node swelling [1]. CD has two clinical subtypes; localized unicentric (UCD) or systemic multicentric (MCD) form and three pathological variants; hyaline vascular (HV), plasma cell (PC), or mixed plasmablastic. The condition often develops in the neck, mediastinum, and pulmonary lymph nodes with rare primary extra-nodal affection [2,3]. Eighteen cases of primary hepatic CD have been reported in the literature, including the present case. Here we report a rare case of primary hepatic UCD associated with hepatocellular carcinoma (HCC) developed after direct-acting antiviral agents (DAAs) and a brief report of the clinicopathological features.

The work has been reported in line with the SCARE 2018 criteria [4].

## 2 Presentation of case

**Fig. 1:**
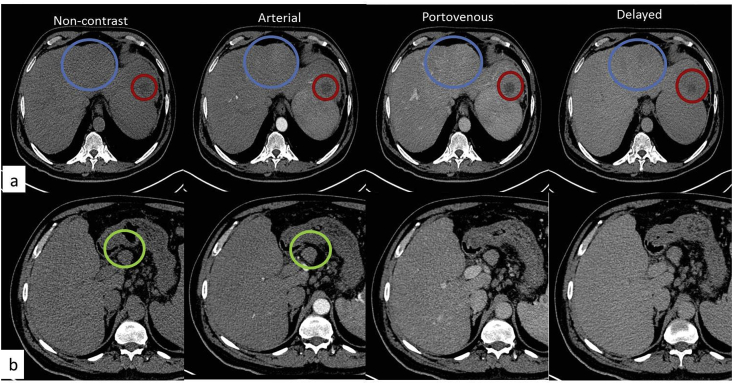
Multiphasic contrast-enhanced CT of liver masses and lymph nodes: (a) axial images at the level of liver showing segment IV two arterially enhancing lesions with delayed washout (typical criteria of HCC) (blue circles), another segment II hypodense non-enhancing lesion through all phases (red circles). (b) Axial images at the level of prominent porta hepatis lymph node (yellow circle) with the absence of hyperenhancement are characteristic features of the primary CD of lymph nodes. (For interpretation of the references to colour in this figure legend, the reader is referred to the Web version of this article.)

A 62 years old male, with no clinical relevant, medical, surgical or family history, received DAAs for chronic hepatitis C virus (HCV) related liver cirrhosis. Three months later, the patient achieved a sustained virologic response (SVR) and stuck to the HCC screening program. Unfortunately, the patient developed three hepatic focal lesions six months after the end of treatment. The patient referred to the oncology unit (multidisciplinary consultants team formed of oncologist, hepatic surgeon, radiologist and pathologist) for further evaluation. Laboratory tests: AST/ALT was 85/46 U/L, total bilirubin/direct bilirubin was 1/0.3 mg/dl, albumin was 3.8 g/dl, Hb was 10.6 g/dl, CRP was 2.8 mg/L, and INR was 1.43. Serum tumor markers were of average values; AFP was 1.7 ng/mL, CEA was 4.1 ng/mL, and CA19.9 was 15 U/mL. In the meantime, the patient underwent triphasic computed tomography (CT) scans of the abdomen and pelvis and revealed a cirrhotic liver with segment IV two hepatic focal lesions. Each measured 1 cm on the greatest dimension displaying typical criteria for HCC. Meanwhile, another large segment II mass measuring 5 cm on size showed a non-enhancing pattern throughout all phases, Fig. 1 (a). The patient was informed by the surgical procedures and signed a written consent. Abdominal exploration was done by a team of hepatobiliary consultant surgeons under supervision of a professor of hepatic surgery. The decision was left lateral segmentectomy of the suspicious mass and subsequent intra-operative ethanol injection of HCC masses based on the clinical and radiological data. Following the operation, patient was compliant and adherent to post-operative instructions regarding pharmacological treatment and avoidance of heavyweight lifting.

**Fig. 2:**
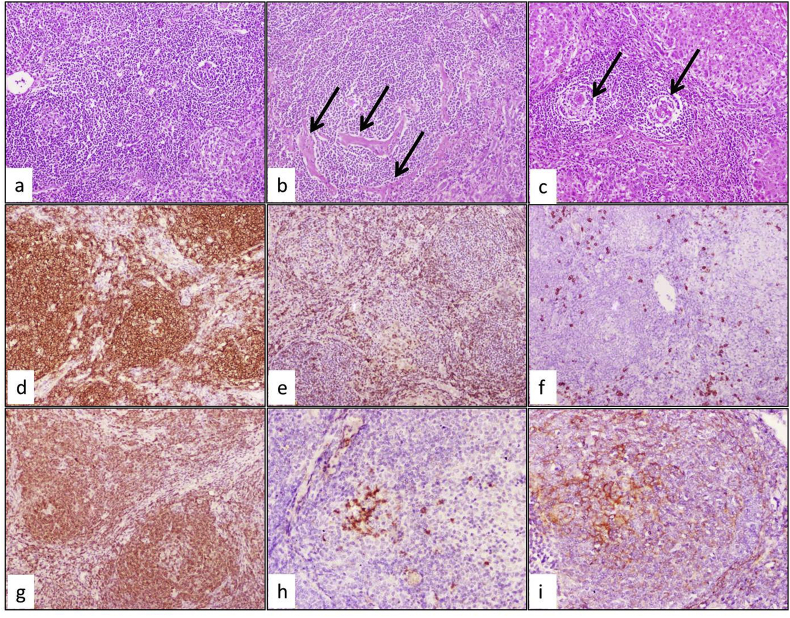
Pathological and immunohistochemical features of hepatic UCD: a) A fairly circumscribed mass formed of hyperplastic lymphoid follicles with atrophic GC and surrounded by concentric rings of small lymphocytes (H&E ×40). B) Proliferating blood vessels with perivascular fibrosis (arrows) (H&E ×200). c) Adjacent non-tumor liver tissue showed features of CD in portal tracts (arrows) (H&E ×40). d) CD20 was diffusely positive in the proliferated lymphoid follicles (IHC ×100). e) CD3 stained for interfollicular T cells (IHC ×100). f) CD138 highlighted the scattered plasma cells (IHC ×100). g) BCL2 stained the expanded mantle zone (IHC ×100). h) while CD10 showed the atrophic GC (IHC ×100). i) EGFR stained for activated FDC (IHC ×200).

**Fig. 3:**
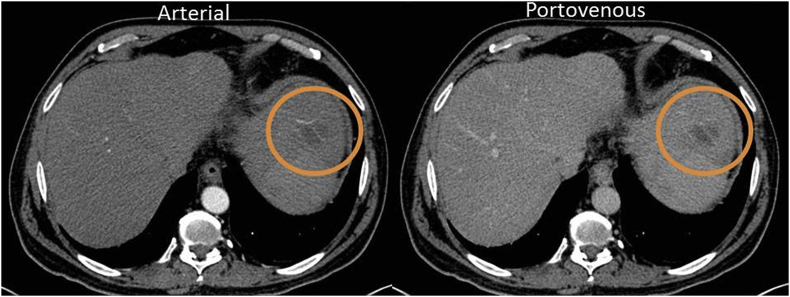
Axial CT images arterial and Porto-venous phases showing segment II non-enhancing lesion with vessels traversing through the lesion with no attenuation or occlusion (orange circles). (For interpretation of the references to colour in this figure legend, the reader is referred to the Web version of this article.)

Postoperative surgical resection revealed a whitish, circumscribed, fleshy mass arising on top of well-established liver cirrhosis. Microscopic examination demonstrated a fairly circumscribed mass formed entirely of lymphocytes arranged in a nodular pattern. The nodular pattern formed of irregular hyperplastic lymphoid follicles of variable sizes surrounding by concentric rings of small lymphocytes represented expanding mantle zones “onion skin”, Fig. 2 (a). These follicles had atrophic germinal centers (GC), occasionally two or more GCs (twinning), and numerous follicular dendritic cells (FDCs). High endothelial venules with perivascular fibrosis radially penetrated the GCs, forming a lollipop structure, Fig. 2 (b). The background liver showed well-established cirrhosis with portal tracts lymphoid follicles showed CD features, Fig. 2 (c). Immunohistochemistry revealed CD20 positive lymphoid follicles with few CD3 interfollicular T-cells, Fig. 2 (d&e). Few plasma cells dispersed in the interfollicular areas stained for CD138, Fig. 2 (f). BCL2 highlighted the expanded mantle zone while CD10 showed the atrophic GC, Fig. 2 (g&h). Epidermal growth factor receptor (EGFR) stained the activated FDC in the GC, Fig. 2 (i). The possibility of associated Hodgkin's lymphoma was excluded by the abstinence of Reed Sternberg (RS) cells in routine staining and negativity for CD15 and CD30 antibodies. Re-evaluation of the lymph nodes by triphasic CT showed prominent non-enhancing porta hepatis lymph nodes. The imaging criteria were suggestive of reactive process commonly associated with liver cirrhosis and excluded the diagnosis of a primary CD of lymph nodes, Fig. 1 (b). A new radiologic finding was observed in the present case in the form of vessels traversing through the lesion with no attenuation or occlusion, Fig. 3. The patient diagnosed as primary, hepatic HV-UCD. The patient was alive for 40 months after resection with disease-free survival proven by serum AFP levels and CT. No further medical or surgical intervention was applied.

## 3 Discussion

Castleman's disease is a primary lymphoproliferative disease affecting lymph nodes with rare reported cases in the extra-nodal site [3]. CD is classified clinically into UCD occurs as a solitary, asymptomatic focal lesion mostly of HV variant and a systemic MCD of possible PC variant. The pathologically HV variant represents 90% of cases and demonstrates atrophic GC with a concentric ring of mantle zone lymphocytes and hyalinized blood vessels within and in between the follicles. The PC variant represents 10% of cases defined by enlargement of lymphoid follicles with an abundance of mature plasma cells, but a lower degree of angiogenesis [5].

Eighteen cases of hepatic CD have been reported in the literature, including the present one. Most cases showed a female predominance (14/18) with a median age of 48 years old. Only a case was reported in a pediatric patient [6]. The prevalence of the female gender could highlight the possible autoimmune pathogenic mechanism of CD [7]. Most hepatic CDs were unicentric (16/18) and presented by abdominal pain or even asymptomatic. However, two cases were of MCD and complained of fever, chills, and easy fatigability. Both symptomatic MCD cases occurred due to a hepatic relapse of nodal CD [8,9]. Different hepatic anatomical localization was encountered, including porta hepatis (7 cases), right lobe (5 cases), caudate lobe (2 cases), hepatoduodenal ligament (one case) and left lobe (the present case).

The pathogenesis of CD is not clear with several viruses that have been implicated in the pathogenesis of nodal CD [5,10]. A defect in the immune regulation and low-grade chronic inflammation induces lymphoid follicles hyperplasia and proliferation of endothelial cells [11]. The etiological association of HCV with nodal CD and the subsequent therapeutic role of interferon regimen have been postulated [12]. However, 16 hepatic CD cases showed no definite etiological association. One case linked to chronic HBV and the present case developed six months after receiving DAAs treatment for HCV [12]. The mechanism of CD in the present case isn't well elucidated. A concomitant HCC and the cirrhotic background might indicate genetic defect and immunological response, which could be accentuated by the administration of DAAs. However, a previous study reported the beneficial effect of DAAs therapy in enhancing anti-CD20 in treating MCD in HCV patients [11]. Therefore, further studies are recommended to assess the actual impact of DAAs therapy in patients with CD.

There were no specific radiological features have been proposed for hepatic CD. Contrast-enhanced CT showed a hypervascular pattern in ten cases, hypovascular in one case, and non-enhancing pattern in another case. The persistent enhancement pattern during the arterial and venous phases could be attributed to the narrowing of the arterial lumen and thickened fibrotic wall [13,14]. Besides, a characteristic calcification and strip-like area were reported in three cases and one case, respectively [15]. However, the present case exhibited no enhancement in all CT phases, with no calcifications or strip-like areas. A new radiologic finding was observed in the form of vessels traversing through the lesion with no attenuation or occlusion, necessitating further studies.

Surgical resection and pathological evaluation remain the diagnostic method in the assessment of hepatic CD. Hepatic resection was performed in 16 out of 18 cases as a diagnostic and therapeutic tool. In the meantime, steroid therapy was done in two pathological approved CD cases [9,16]. The prognosis of the hepatic CD is excellent, with all hepatic CD cases showing disease-free survival after therapy.

## 4 Conclusions

Despite the hepatic CD is a rare entity; we should put it in the differential diagnosis of a hepatic focal lesion with atypical radiological findings. The abstinence of characteristic clinical and radiological features makes pathological evaluation is the gold standard in hepatic CD diagnosis. The role of DAAs in the prevention or progression of hepatic CD necessitates further studies.

## Provenance and peer review

Not commissioned, externally peer reviewed.

## Ethical approval

Not required, all was done on the seek of diagnosis and proper management of the patient.

## Sources of funding

No funding.

## Author contribution

All authors of this paper have participated in its drafting and approved the final version submitted. DS, HA, YF, and SK wrote the case report. TH, NE, DE, and DS contributed in the pathological diagnosis. YF contributed in the surgical part. HA provided the radiological imaging and interpretation of data. SK shared in the oncology section. AM performed the technical immunohistochemical work. MS and DS interpreted the immunohistochemical work. DS, NE, TH revised, and edited the final version.

## Registration of research studies

1. Name of the registry: Not applied.
2. Unique Identifying number or registration ID:
3. Hyperlink to your specific registration (must be publicly accessible and will be checked):

## Guarantor

Dina Sweed.

## Consent

Written informed consent was obtained from the patient for publication of this case report and accompanying images. A copy of the written consent is available for review by the Editor-in-Chief of this journal on request.

## Declaration of competing interest

All authors have no conflicts of interest to declare.
