# Supplementary material for: Post direct acting anti-viral agents associated primary hepatic Castleman's disease: A case report
Source: Ann Med Surg (Lond). 2020 Aug 23;58:37–40. doi: 10.1016/j.amsu.2020.08.019 (PMC7475233; doi:10.1016/j.amsu.2020.08.019)
Supplement: Multimedia component 1 [file mmc1.docx]

| SCARE 2018 Checklist | | | |
| --- | --- | --- | --- |
| Topic | Item | Checklist item description | Page Number |
| Title | 1 | The words “case report” should appear in the title. The title should also describe the area of focus (e.g. presentation, diagnosis, surgical technique or device or outcome). | Yes |
| Key Words | 2 | 3 to 6 key words that identify areas covered in this case report (include "case report" as one of the keywords). | Yes |
| Abstract | 3a | Introduction — Describe what is unique or educational about the case (i.e. what does this work add to the surgical literature, and why is this important?). | Page 2  Lines 31-35 |
|  | 3b | Presenting complaint and investigations – describe the patient's main concerns and important clinical findings. | Page 2  Lines 25-27 |
|  | 3c | The main diagnoses, therapeutics interventions, and outcomes. | Page 2  Lines 27-31 |
|  | 3d | Conclusion — Describe the main lessons to “take-away” from this case study | Page 2  Lines 31-35 |
| Introduction | 4 | Background – summarise what is unique or educational about the case. Give reference to the relevant surgical literature and current standard of care. The background should be referenced, and 1-2 paragraphs in length. | Page 3  Lines 37-46 |
| Patient Information | 5a | Demographic details – include de-identified demographic details on patient age, sex, ethnicity, occupation. Where possible, include other useful pertinent information e.g. body mass index and hand dominance. | Page 3  Lines 48-51 |
|  | 5b | Presentation - describe the patient’s presenting complaint (symptoms). Describe the patient’s mode of presentation (brought in by ambulance or walked into Emergency room or referred by family physician). | Page 3  Lines 50-53 |
|  | 5c | Past medical and surgical history, and relevant outcomes from interventions | Page 3  Lines 48-49 |
|  | 5d | Other histories – Describe the patient’s pharmacological history including allergies, psychosocial history (Drug, smoking, and if relevant, accommodation, walking aids), family history including relevant genetic information. | Page 3  Lines 48-49 |
| Clinical Findings | 6 | Describe the relevant physical examination and other significant clinical findings. Include clinical photographs where relevant and where consent has been given. | NA |
| Timeline | 7 | Inclusion of data which allows readers to establish the sequence and order of events in the patient's history and presentation (using a table or figure if this helps). Delay from presentation to intervention should be reported. | Pages 3-4  Lines 48-88 |
| Diagnostic Assessment | 8a | Diagnostic methods – describe all investigations taken to arrive at methods: physical exam, laboratory testing, radiological imaging, histopathology. | Page 3  Lines 67-87 |
|  | 8b | Diagnostic challenges – describe what was challenging about the diagnoses, where applicable, for example access, financial, cultural. | NA |
|  | 8c | Diagnostic reasoning – Describe the differential diagnoses and why they were considered. | Page 3  Lines 80-83 |
|  | 8d | Prognostic characteristics when applicable (e.g. tumour staging or for certain genetic conditions). Include relevant radiological or histopathological images in this section. | Page 3  Lines 80-83 |
| Therapeutic Intervention | 9a | Pre-intervention considerations – if there were patient-specific optimisation measures taken prior to surgery or other intervention these should be included e.g. treating hypothermia/hypovolaemia/hypotension in a burns patient, Intensive care unit treatment for sepsis, dealing with anticoagulation/other medications, etc. | NA |
|  | 9b | Interventions – describe the type(s) of intervention(s) deployed (pharmacologic, surgical, physiotherapy, psychological, preventive). Describe the reasoning behind this treatment offered. Describe any concurrent treatments (antibiotics, analgesia, anti-emetics, nil by mouth, Venous thrombo-embolism prophylaxis, etc). Medical devices should have manufacturer and model specifically mentioned. | Page 3  Lines 61-64  Page 4  Line 88 |
|  | 9c | Intervention details – describe what was done and how. For surgery include details on; anaesthesia, patient position, use of tourniquet and other relevant equipment, prep used, sutures, devices, surgical stage (1 or 2 stage, etc). For pharmacological therapies include information on the formulation, dosage, strength, route, duration, etc. Include intra-operative photographs and/or video or relevant histopathology in this section. Degree of novelty for a surgical technique/device should be mentioned e.g. "first in human". | Page 3  Lines 61-64 |
|  | 9d | Who performed the procedure - operator experience (position on the learning curve for the technique if established, specialisation and prior relevant training). For example, “junior resident with 3 years of specialised training” | Page 3  Lines 62-63 |
|  | 9e | Changes – if there were any changes in the interventions, describe these details with the rationale. | NA |
|  | 9f | Post intervention consideration: post-operative instruction and place of care | Page 3  Lines 65-67  Page 4  Lines 88-89 |
| Follow-up and  Outcomes | 10a | Follow-up – describe 1) When the patients was followed up. 2) Where. 3) How (imaging, tests, scans, clinical examination, phone call), and 4) whether there were any specific post-operative instructions. Future surveillance requirements - e.g. imaging surveillance of endovascular aneurysm repair or clinical exam/ultrasound of regional lymph nodes for skin cancer. | Page 4  Lines 88-89 |
|  | 10b | Outcomes - Clinician assessed and (when appropriate) patient-reported outcomes (e.g. questionnaire details). Relevant photographs/radiological images should be provided e.g. 12 month follow-up. | Page 4  Lines 88-89 |
|  | 10c | Intervention adherence/compliance - where relevant how well patient adhered to and tolerated their treatment. For example, post-operative advice (heavy lifting for abdominal surgery) or tolerance of chemotherapy and pharmacological agents | Page 3  Lines 66-67 |
|  | 10d | Complications and adverse events – all complications and adverse or unanticipated events should be described in detail and ideally categorised in accordance with the Clavien-Dindo Classification. How they were prevented, diagnosed and managed. Blood loss, operative time, wound complications, re-exploration/revision surgery, 30-day post-op and long-term morbidity/mortality may need to be specified. If there were no complications or adverse outcomes this should also be included. | NA |
| Discussion | 11a | Strengths – describes the strengths of this case | Pages 4-5Lines 125-130 |
|  | 11b | Weaknesses and limitations in your approach to this case. For new techniques or implants - contraindications and alternatives, potential risks and possible complications if applied to a larger population. If relevant, has the case been reported to the relevant national agency or pharmaceutical company (e.g. an adverse reaction to a device) | NA |
|  | 11c | Discussion of the relevant literature, implications for clinical practice guidelines and any relevant hypothesis generation. | Pages 4-5  Lines 99-133 |
|  | 11d | The rationale for your conclusions. | Page 5  Lines 132-138 |
|  | 11e | The primary “take-away” lessons from this case report. | Page 5  Lines 134-138 |
| Patient Perspective | 12 | When appropriate the patient should share their perspective on the treatments they received. | Page 4  Lines 61-62 |
| Informed Consent | 13 | Did the patient give informed consent for publication? Please provide if requested by the journal/editor. If not given by the patient, explain why e.g. death of patient and consent provided by next of kin or if patient/family untraceable then document efforts to trace them and who within the hospital is acting as a guarantor of the case report. | yes |
| Additional Information | 14 | Conflicts of Interest, sources of funding, institutional review board or ethical committee approval where required. | yes |
